# The Accelerated Aging Impact on Mechanical and Thermal Properties of Polypropylene Composites with Sedimentary Rock Opoka-Hybrid Natural Filler

**DOI:** 10.3390/ma15010338

**Published:** 2022-01-04

**Authors:** Paulina Jakubowska, Grzegorz Borkowski, Dariusz Brząkalski, Bogna Sztorch, Arkadiusz Kloziński, Robert E. Przekop

**Affiliations:** 1 Institute of Technology and Chemical Engineering, Faculty of Chemical Technology, Poznan University of Technology, Berdychowo 4, 60-965 Poznan, Poland; arkadiusz.klozinski@put.poznan.pl; 2 Faculty of Geographical and Geological Sciences, Adam Mickiewicz University in Poznan, B. Krygowskiego 10, 61-680 Poznan, Poland; grzegorz.borkowski@amu.edu.pl; 3 Faculty of Chemistry, Adam Mickiewicz University in Poznan, Uniwersytetu Poznanskiego 8, 61-614 Poznan, Poland; dariusz.brzakalski@amu.edu.pl; 4 Centre for Advanced Technologies, Adam Mickiewicz University in Poznan, Uniwersytetu Poznanskiego 10, 61-614 Poznan, Poland; bogna.sztorch@amu.edu.pl

**Keywords:** weathering, UV blocker, stabilizer, thermoplastics, polyolefin, degradation, biofiller, biomaterials, Opoka, sedimentary rock, hybrid filler

## Abstract

This paper presents the impact of accelerated aging on selected mechanical and thermal properties of isotactic polypropylene (iPP) composites filled with sedimentary hybrid natural filler-Opoka rock. The filler was used in two forms: an industrial raw material originating as a subsieve fraction natural material, and a rock calcinated at 1000 °C for production of phosphorous sorbents. Fillers were incorporated with constant amount of 5 wt % of the resulting composite, and the material was subjected to accelerated weathering tests with different exposition times. The neat polypropylene and composites with calcium carbonate as a reference filler material were used for comparison. The aim of the research was to determine the possibility of using the Opoka rock as a new hybrid filler for polypropylene, which could be an alternative to the widely used calcium carbonate and silica. The thermal, mechanical, and structural properties were evaluated by means of differential scanning calorimetry (DSC), tensile tests, scanning electron microscopy (SEM), and Fourier-transform infrared spectroscopy with attenuated total reflectance (FTIR/ATR) prior to and after accelerated aging. As a result, it was found that the composites of polypropylene with Opoka were characterized by similar or higher functional properties and higher resistance to photodegradation compared to composites with conventional calcium carbonate. The results of measurements of mechanical properties, structural and surface changes, and the carbonyl index as a function of accelerated aging proved that Opoka was an effective ultraviolet (UV) stabilizer, significantly exceeding the reference calcium carbonate in this respect. The new hybrid filler of natural origin in the form of Opoka can therefore be used not only as a typical powder filler, but above all as a UV blocker/stabilizer, thus extending the life of polypropylene composites, especially for outdoor applications.

## 1. Introduction

With the development of industrial technologies, the demand for polymer materials continues to increase. Production of polymer-based materials requires mining and extracting petroleum resources [1], and during their service life, these products are degraded and emit harmful or environmentally burdening products. Increasing pollution of the natural environment and depletion of global oil and coal deposits force the industry to search for and develop new, more sustainable, and low-energy polymer materials [2,3,4]. This goal can be achieved through the use of biopolymers and biodegradable materials (e.g., bio-polyethylene (BPE) [5], poly(3-hydroxybutyrate-co-3-hydroxyvalerate) (PHBV) [6], polylactide (PLA) [7]), and/or the production of polymer composites with fillers of natural origin using, for example, natural fibers [8,9,10], lignin [3,11], or natural powder fillers [12,13,14].

Due to its very good mechanical properties, resistance to water and a broad range of chemicals, excellent processability, and low price, polypropylene (PP) is one of the most popular and widely used thermoplastics [15,16,17,18]. Among its greatest disadvantages is the gradual deterioration of the properties occurring during exposure to environmental conditions, such as UV radiation, varying temperature (especially freezing–thawing cycles), oxygen, and moisture, which is referred to as aging [15,19,20,21,22,23]. Oxygen affects polymers already during their conversion into specific goods during polymer processing, as well as during their use; additionally, ozone is highly corrosive to all organic materials when applied as a sterilizing agent or existing as tropospheric ozone. This action of different forms of oxygen, along with other atmospheric factors, promotes the formation of free radicals, which ultimately, through the degradation process, cause changes in the molecular and macromolecular structure, and cross-linking of polymers [20]. The degradation causes the deterioration of the chemical, physical, and mechanical properties of polymeric materials, and thus significantly shortens their service life [21,22].

UV radiation causes photodegradation of polymers and is one of the most destructive factors in polymer weathering. According to Singh B. and Sharma N. [24], photodegradation of polyolefins in the presence of oxygen is an autocatalytic process. This process begins with the absorption of UV photons by catalyst residues, chromophores, protic groups, and molecules/polymer chains containing unsaturated bonds, carbonyl groups, and/or aromatic rings. Further exposure to UV radiation changes the molecular structure of the polymer, which leads to the typical signs of aging such as peeling, cracking, migration, discoloration, and changes in mechanical properties [23,25,26]. Due to the limited ability of UV radiation to penetrate and of oxygen to diffuse through the polymer, the degree of PP photodegradation decreases, as the harmful factors need to penetrate deeper into the surface of the material. In the case of a polymer being exposed to elevated temperature in the presence of oxygen, the main mechanism of its damage is thermo-oxidation, which is a combination of surface degradation and formation of microcracks that spread through the structure of the material [27,28]. It should be emphasized that thermo-oxidation can take place in the entire volume of the product, unlike photo-oxidation, which takes place mainly on the irradiated surface.

Due to the presence of a hydrogen atom on the tertiary carbon atom and its ease of being abstracted to form hydroperoxides, which induce the chain scission of PP, the degradation of polypropylene used in the natural environment occurs very easy [17,29]. In order to slow down the degradation of polypropylene, at the stage of its processing, special modifiers are introduced, such as UV stabilizers, antioxidants, and/or various types of fillers [16,20]. These agents stabilize the material properties of the product and increase its service life. The influence of fillers on the degradation of polymer composites has been discussed in many publications [16,25,27,30,31,32,33,34,35], taking into account that the natural fillers limiting photo- and thermo-oxidation of polymers include, e.g., lignin [30], sisal [32], ground chestnut shells [33], and jute [34]. On the other hand, the additives such as calcium carbonate [31] and silica [16,35], the influence of which on polymer degradation depends on the weight content thereof in the composite, the size of the filler grain and its surface modification are recognized.

Due to its low cost and good availability, calcium carbonate is one of the most commonly used inorganic fillers [25,31,36,37]. The addition of calcium carbonate to a polypropylene matrix improves the thermal stability and impact strength, and enhances the mechanical parameters (flexural strength, elastic modulus) of the resulting composites [36,38,39]. The modification of polypropylene by adding silica also improves its temperature stability [40]. The silica addition reinforces polypropylene composites to be subjected to loading under static tension conditions [41].

Opoka is a mineral material of natural origin with potential use as a novel hybrid filler for polymers. Opoka is a sedimentary rock, being a transitional form between carbonate and siliceous rocks, formed in the Upper Cretaceous period as a result of the decalcification of primary rocks [42], and was extensively characterized in our previous work [43]. The content of silica (SiO_2_) in Opoka rock ranges from 5 to 75 wt %, and it occurs predominantly in the form of opal, chalcedony, or quartz. Depending on their calcium content (as calculated for CaO) or silica content, Opokas can be divided into heavy Opoka grades, in which carbonate contents predominate, and light Opoka grades, also known as decalcified Opoka, with silica predominating in their composition [42,44,45].

Owing to their thermal and mechanical properties, Opoka finds application in the building industry, cement production, road construction, and the manufacture of decorative lining and ceramic materials [42,46]. The sorption properties of Opoka materials are utilized in the processes of water and sewage purification to remove phosphorus, magnesium, and iron, among others [43,47].

Due to the content of both calcium carbonate and silica in the chemical composition of Opoka, as well as its interesting physicochemical properties [43,48], the aim of this study was to determine the influence of accelerated aging on the thermal and mechanical properties of polypropylene composites filled with Opoka as a hybrid natural filler. This was a continuation of our study on Opoka/iPP composites, in which the mechanical properties of the obtained composites and the processability (compounding and dispersion) of the filler were covered previously.

## 2. Materials and Methods

### 2.1. Materials

Isotactic polypropylene (iPP) Moplen HP456J (Basell Orlen Polyolefins, Płock, Poland) with a density of 0.900 ± 0.003 g/cm^3^ and a melt flow index (MFI) (230°; 2.16 kg) = 3.09 ± 0.04 g/10 min was used as the matrix of the composites. The fillers used in the tests were: calcium carbonate (CaCO_3_) Omyacarb 2-VA (Omya, Warsaw, Poland), with an average grain size (d50) of 3.86 µm, and two types of Opoka: first in the form of industrial raw material originating (as a subsieve fraction) from the production of sorbents used in water-treatment processes; and second in the form of waste rock calcinated at 1000 °C for production of ceramics. The raw Opoka consisted of CaCO_3_/SiO_2_, and the calcinated Opoka of CaO/SiO_2_ [43]. The samples were named in reference to the filler type. Both Opoka samples were ground (72 h) and sieved through a vibrating screen with a mesh size of 40 µm to eliminate the particle fraction above this size. The average grain size (d50) of the Opoka samples was 28.6 µm and 29.4 µm, respectively. Detailed description of physicochemical properties of the filler was presented in our previous work [43].

### 2.2. Sample Preparation

The polymer composites containing 5 wt % of the filler were manufactured using iPP as the matrix. The composites were produced in a two-step process. In the first stage, polypropylene was rolled together with a given filler on a two-roll mill (Zamak Mercator, Skawina, Poland). Then, iPP masterbatches containing 50 wt % of fillers were obtained, and subsequently diluted to a concentration of 5 wt % in the process of extrusion with cold granulation. For this purpose, a Zamak Mercator twin-screw extruder (Zamak Mercator, Skawina, Poland) with a screw diameter of d = 16 mm and an L/D ratio of 40 were used. Samples for accelerated aging properties testing were obtained in the injection-molding process using a Battenfeld PLUS 35/75 hydraulic injection-molding machine (Battenfeld, Vienna, Austria) with the following parameters: injection die temperature 225 °C, mold temperature 25 °C, cooling time 20 s, p_1_ = 1200 bar, p_2_ = 500 bar. The obtained samples were in the form of standardized testing dumbbells in accordance with ISO 527-2:2012: Plastics—Determination of tensile properties—Part 2: Test conditions for moulding and extrusion plastics (type 1A). Composites were named as follows: iPP/CaCO_3_–iPP/CC; iPP/CaCO_3_/SiO_2_–iPP/RO (raw Opoka); and iPP/CaO/SiO_2_–iPP/CO (calcinated Opoka).

### 2.3. Accelerated Aging Process

All samples were subjected to an accelerated aging process using QLAB QUV accelerated weathering tester according to ISO 4892-3:2016: Plastics—Methods of exposure to laboratory light sources—Part 3: Fluorescent UV lamps (Q-LAB Corporation, Westlake, OH, USA). A 1000 h aging process was conducted under 0.76 W/m^2^ UV light irradiance at 340 nm wavelength, with a cyclic temperature ranging from 60 °C during 8 h dry UV light exposure period to 50 °C at 4 h in the water spray phase. The samples were taken for testing after 250, 500, 750, and 1000 h, and assigned with an index consistent with the time spent in the aging chamber; e.g., PP/RO_750 was a composite containing 5 wt % of the raw Opoka after 750 h of accelerated aging.

### 2.4. Analytical Methods

Differential scanning calorimetry (DSC) was performed using a NETZSCH model DSC-200 (Erich NETZSCH GmbH & Co. Holding KG, Selb, Germany) with computer software for raw data analysis. The measurements were applied to the samples weighing 7–7.5 mg, taken from the central parts of the injection-molded specimens of a standard dumbbell shape. The examination took place in a temperature range of 25–230 °C under an argon atmosphere. All measurements were performed according to the following program: heating from 25 to 230 °C at a scanning rate of 10 °C/min, keeping the sample at 230 °C for 2 min, and then cooling down from 230 °C at a scanning rate of 5 °C/min. Two cycles were performed to eliminate the thermal/processing memory of the samples (the first heating–cooling cycle), and both cycles were analyzed to fully define the thermal properties of the composites. Aluminum pans were used, with an empty pan serving as a reference. In order to determine the effects of used hybrid fillers and the accelerated aging process on the thermal properties of the polymer matrix, both the reference sample and all composites were tested under the same conditions (according to the above-mentioned temperature program).

The following crystallization and melting parameters were determined: melting temperature (*T_m_*), melting enthalpy (Δ*H_m_*), crystallization temperature (*T_c_*), and degree of crystallinity (*X_c_*). The degree of crystallinity of the neat iPP and its composites was calculated using following equation:

(1) $$X_{c}=\frac{\Delta H_{m}}{\left(1-\varphi \right)\Delta H_{m}^{0}}$$

where ∆*H_m_* is the melting enthalpy (J/g), *φ* is the weight % of the filler in the composite (%), and $\Delta H_{m}^{0}$ is the mean melting enthalpy of fully crystalline iPP, which equals 207 J/g [20,49].

Mechanical properties under static load conditions such as tensile modulus (Et), tensile strength (σM), and elongation at break εB of the samples were evaluated by means of the static tensile test, according to the ISO 527-2 standard. A ZwickRoell Z020TH AllroundLine universal testing machine (ZwickRoell GmbH & Co. KG, Ulm, Germany) was used. The traverse speed was set to 1 mm/min during the determination of the tensile modulus and 50 mm/min during the remaining part of the test. Shore hardness (D scale) was measured with a ZwickRoell durometer, according to the ISO 868:2005 standard: Plastics and ebonite—Determination of indentation hardness by means of a durometer (Shore hardness).

Infrared absorption spectra with Fourier transformation (ATR-FTIR) was performed using a NICOLET 5700 instrument (Thermo Fisher Scientific, Waltham, MA, USA). All measurements were performed in the wavenumber range of 4000 to 600 cm^−1^ with 64 scans.

The surface morphology, structure, and composition of the composites was analyzed and imaged with a scanning electron microscope (QUANTA 250 FEG, Poznan, Poland) operated under high-vacuum conditions at a 5 kV acceleration voltage, and in secondary electron imaging mode. The energy-dispersive spectroscopy (EDS) analyses were conducted at a beam acceleration voltage of 15 kV using an EDAX Octane SDD detector. EDS maps of element overlay were made at a resolution of 0.3 µm.

## 3. Results and Discussion

### 3.1. Differential Scanning Calorimetry

Table 1 presents the results of DSC measurements, including melting and crystallization temperature (*T_m_* and *T_c_*, respectively), melting enthalpy (∆*H_m_*), and crystallinity (*X_c_*) determined for the neat iPP and composites thereof with the studied fillers. Crystallinity was calculated using Formula (1).

When comparing the obtained *T_m_* and *T_c_* values, it was noticed that the introduction of calcium carbonate and both types of rocks into the polypropylene matrix did not significantly change the melting point of the composites. Similar results for the iPP composites with CaCO_3_ and SiO_2_ were reported in [50,51]. This is typical for systems in which there is no interaction between the polymer and the filler surface, and the filler does not affect the size of the polymer spherulites formed during the crystallization of the material and influencing the *T_m_* [52,53]. On the other hand, the applied fillers increased the crystallization temperature of composites due to the nucleating nature of the filler. The nucleating properties of the fillers used were confirmed by the determined increase in the crystallinity index *X_c_*. As a function of the filler used, the increase was as follows: CC < RO < CO, which represented 0.4%, 5.1%, and 5.2%, respectively, after the second melting cycle, in relation to neat PP. Moreover, the *X_c_* value of the unaged samples calculated during the first heating cycle was significantly lower compared to the results obtained in the second cycle. This was due to the low temperature of the mold during injection molding (allowing for a shorter processing cycle, but unfavorable from the point of view of the crystallization of the polymer), resulting in a high cooling rate of the molten polymer, and consequently the solidification of the material in a more amorphous form [33,54]. During the DSC experiment, the crystallization was performed at a low rate, which gave the molecular chain sufficient time to order into the lattice [55]. As the *T_c_* was higher for the obtained composites, it reduced their degree of supercooling. The degree of supercooling is the temperature difference of Tm and the temperature at which crystallization takes place at maximum speed [56]:

(2) $$\Delta T=T_{m}-T_{c}$$

where *T_m_* is the melting temperature of the polymer determined from the DSC curve of the heating (°C), and *T_c_* is the crystallization temperature determined from the DSC curve of the cooling (°C).

The smaller this difference, the faster the crystallization process may be performed during processing, which in turn contributes to the shortening of the manufacturing process (injection molding, extrusion). Satisfactory results for used composites were obtained—the ∆*T* value decreased by 3.7 °C for both the CaCO_3_ and CaCO_3_/SiO_2_ fillers, and by 5.9 °C for CaO/SiO_2_ in relation to the reference sample.

**Figure 1:**
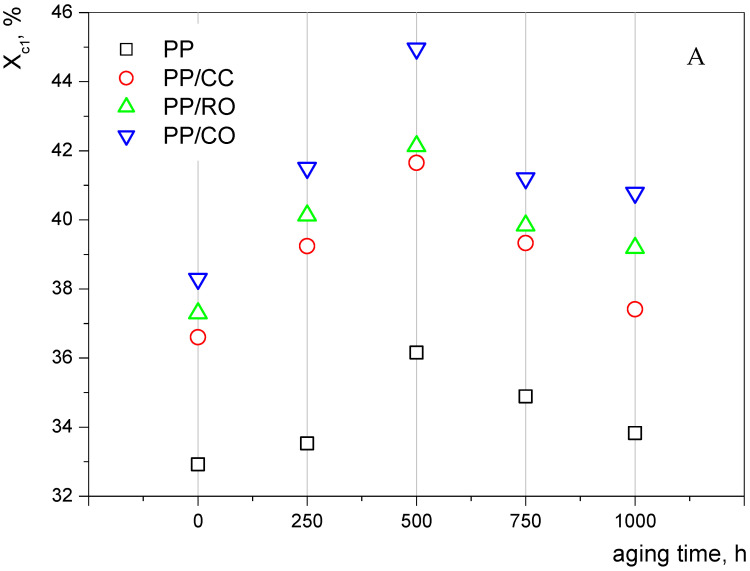

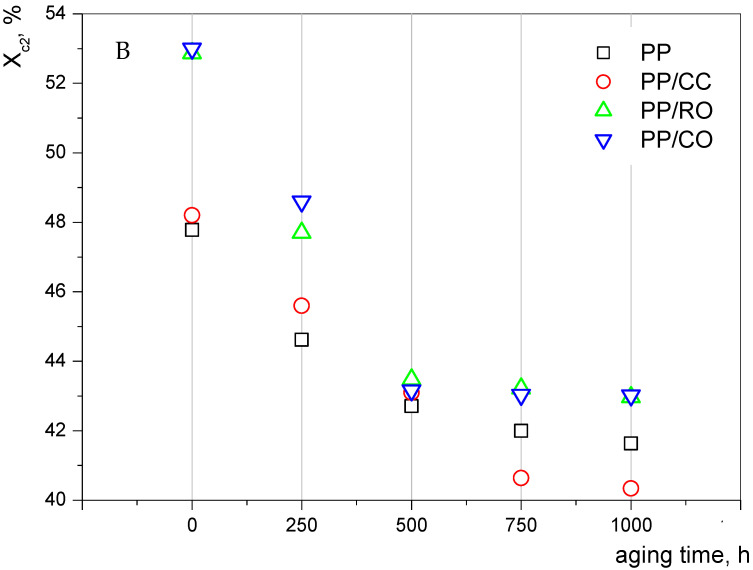
Degree of crystallinity of iPP and its composites, determined by DSC: (**A**)—first heating; (**B**)—second heating.

Table 2 shows the values of the melting and crystallization points of the iPP samples and the composites subjected to the accelerated aging process. For the composite samples containing reference filler and raw Opoka, a significant decrease in *T_m_* and *T_c_* was recorded after 250 h of accelerated aging. In the further period of exposure to UV radiation and temperature, these values fluctuated around a constant value (changed in the range of 1 to 3 °C), and showed neither an increasing nor decreasing trend. A similar result was also observed by Valadez-Gonzalez et al. for HDPE and HDPE/CaCO_3_ composites [36]. On the other hand, a more clear influence of exposure to UV light and cyclic heating was visible in the case of the degree of crystallinity (Figure 1).

Usually the thermal data for thermoplastics is extracted from the second DSC run in order to erase their melt memory. However, extracting data from both cycles allowed for a better understanding of the polymer’s behavior, especially when it came to polymer aging. The results of the first melting cycle will be discussed first. In Figure 1, it can be seen that for the first melting (Figure 1A), the *X_c_* of iPP and composites thereof filled with calcium carbonate and Opoka tended to increase together with aging time up to 500 h. This behavior was attributed to recrystallization due to iPP chain scission, providing more mobility for polymer further crystallization [33,50,57]. As the temperature during accelerated aging was only 60 °C, this rearrangement of the crystalline structure was caused by the simultaneous influence of both heat and UV radiation, inducing partial degradation of the polymer. The freed segments in the amorphous region had sufficient mobility to rearrange into a crystalline phase, increasing the degree of crystallinity during degradation. This process is known as chemicrystallisation [54,55]. Observed increment of the crystallinity could also be attributed to the preferential oxidation of the amorphous phase of the iPP, as well to the formation of new crystallites induced by the chain-scission reactions [36,37]. Above 500 h, the level of polymer degradation was so high that the spherulites underwent decay and the crystallinity dropped. In terms of the studied polypropylene fillers and the function of exposure time to UV radiation, it was noticed that for each aging time, the recorded values of the degree of crystallinity were consistent with the previously presented relationship; i.e., *X_c_* CC < *X_c_* RO < *X_c_* CO. After 1000 h of accelerated aging, the degree of crystallinity of all tested materials was higher than that of unaged samples. It should be emphasized, however, that this increase recorded for composites containing Opoka fillers reached much higher values; i.e., the increase in *X_c_* for PP_1000 in relation to iPP was 0.91%, for iPP/CC 0.81%, for iPP/RO 1.89%, and for PP/CO as much as 2.49%. The hybrid filler used thus reduced the degradation of the polypropylene composites, and could be used not only as a typical filler of natural origin, but also as a UV blocker/stabilizer. From among the two tested hybrid fillers, calcined Opoka showed better stabilizing properties. The most likely factor behind this behavior was the low UV permeability of SiO_2_, resulting in protection of the polymer from the photodegradation, especially toward the deeper fraction of the composite [58,59]. DSC curves during the first heating showed the crystalline structure after aging, whereas DSC curves during the second heating showed the crystallization capability of the aged sample [57]. Speaking of the second melting cycle (Figure 1B), the drop in crystallinity followed a time-dependent trend. This was due to the increased polymer degradation near the surface of the sample. After melting, the entire bulk of the polymer became contaminated with the degraded species, which inhibited proper crystallization and reformation of the previously more or less unaffected spherulites. During exposure to UV radiation and temperature, the degree of crystallinity determined on the basis of the second heating curves for all tested materials gradually decreased. For the neat iPP and PP/CC composites, this decrease was more uniform, while for the Opoka-filled samples, the decrease slowed after 500 h, which hinted that the filler protected mainly the inner volume of the polymer, remaining mostly unaffected during longer exposure; while on the surface, it proceeded more freely, quickly resulting in imparted ability of the polymer to crystallize under recycling. This observation explained the importance of seeking effective UV stabilizers for recyclable semicrystalline thermoplastics.

### 3.2. Mechanical Properties

Before starting the aging tests, the mechanical properties of unaged samples of neat iPP and the composites thereof were characterized, and the obtained results are summarized in Table 3. A detailed analysis of the obtained results was presented in the previous work [43].

The unaged samples were characterized by a slightly increased Young’s modulus, with the highest increase, 15.5%, for the raw Opoka-filled composite, when compared to neat iPP. There was also a decrease in the strain at break of the composites compared to the iPP matrix, by 16.6, 21.0, and 15.0% for iPP/CC, iPP/RO, and iPP/CO, respectively. The observed changes were expected and justified because particles of rigid powder fillers were introduced into the continuous structure of iPP, which, during the stretching process, resisted the movement of polymer elastic segments (the filler being concentrated in an amorphous polymer phase), thus increasing the Young’s modulus and reducing the deformation of the composites [34,35,51]. At the same time, the tensile strength remained virtually unchanged, as no particular reinforcing action was provided by the neutral filler.

**Figure 2:**
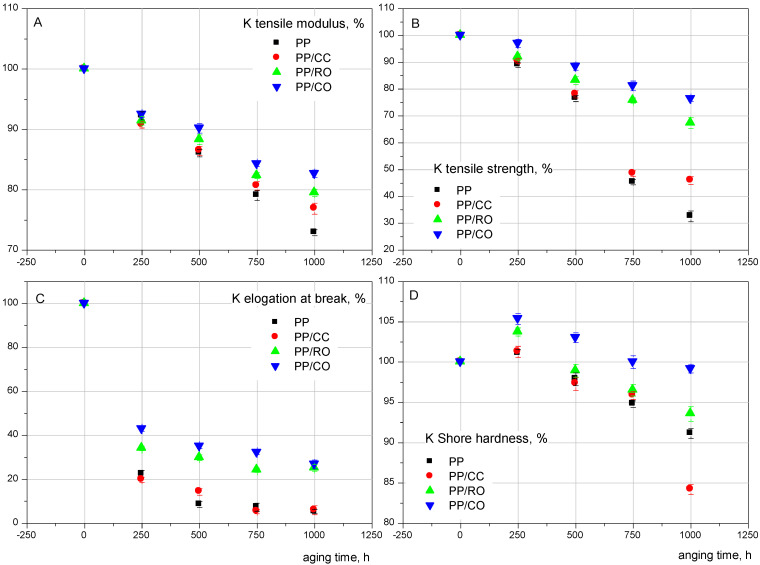
The changes in mechanical properties of iPP and its composites after accelerated aging (K coefficients): (**A**)—Young’s modulus; (**B**)—tensile strength; (**C**)—elongation at break; (**D**)—Shore hardness (D scale).

The results of the tensile properties of the tested materials as a function of the duration of accelerated aging are summarized in Figure 2A–D. To present the results obtained from tensile and hardness testing, the coefficient K was calculated for each composite. This factor represents the percentage change in the mechanical properties over time, and is the ratio of the value obtained for a composite after a specific amount of time to the value of the initial sample.

As the time of exposure to UV radiation increased, the Young’s modulus, tensile strength and elongation at break decreased. Due to the increasing crystallinity discussed earlier, the Shore hardness increased at first, and then dropped with further aging. This behavior was observed for all tested materials. The reduction in properties was due to chain scission and degradation, and the drop in the average molecular weight occurring to iPP during exposure to UV radiation for a longer duration.

**Figure 3:**
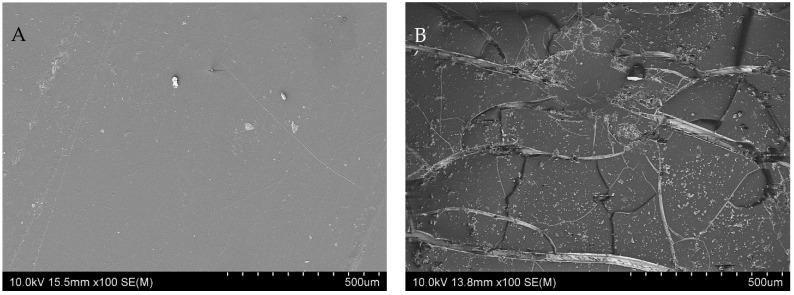
SEM images of (**A**)—unaged iPP and (**B**)—iPP after UV exposure for 1000 h.

In terms of the type of filler type used, it was noticed that changes in mechanical properties caused by aging time accompanied neat iPP and composites containing calcium carbonate to a much greater extent than composites filled with Opoka (the relationship was analogous to the degree of crystallinity). For example, after 1000 h of accelerated aging, E_t_ for neat iPP dropped by 27.1%, for iPP/CC by 23.2%, for iPP/RO by 20.5%, and for iPP/CO by 17.4%. According to [32], the increase in crystallinity during exposure causes shrinkage of the degraded material. The tendency of surface contraction, together with the decrease in average molecular weight, ultimately leads to the formation of surface cracks, even without any applied stress [60]. This is one of the reasons for polymer embrittlement caused by photodegradation, as the surface cracks act as micronotches for material rupture under a mechanical load. The formation of surface cracks in the case of iPP after accelerated aging for 1000 h can be seen in the SEM pictures given in Figure 3A,B.

### 3.3. Microstructure

**Figure 4:**
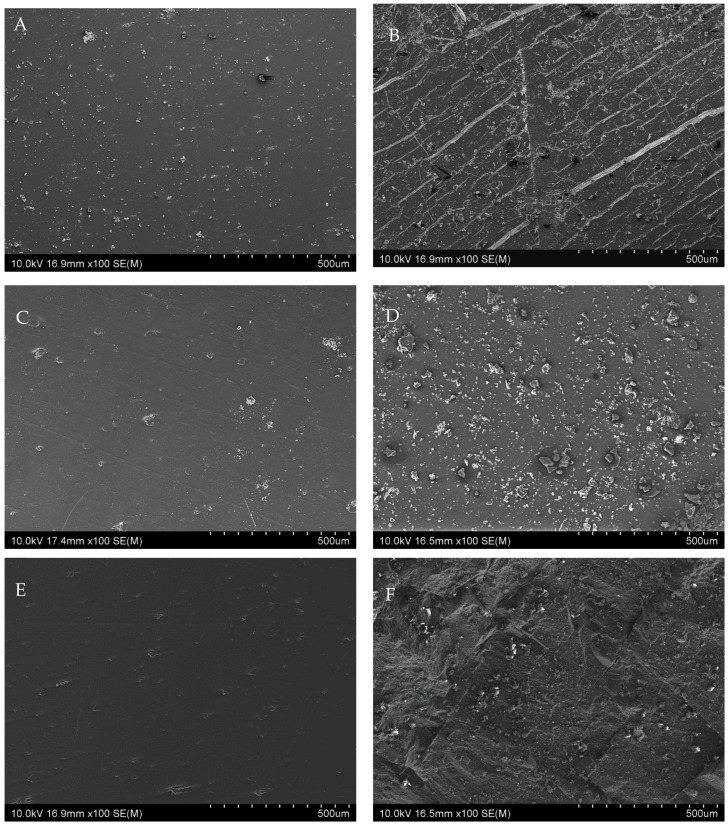
SEM images of: (**A**)—unaged iPP/CC; (**B**)—iPP/CC after UV exposure for 1000 h; (**C**)—unaged iPP/RO; (**D**)—iPP/RO after UV exposure for 1000 h; (**E**)—unaged iPP/CO; (**F**)—iPP/CO after UV exposure for 1000 h.

It can be observed that the neat iPP sample, while smooth before aging (Figure 3A) had many surface microcracks and porosity, and was brittle after 1000 h of exposure to UV radiation (Figure 3B). This also is evidenced in Figure 2B—the tensile strength of the iPP sample after 1000 h exposure to ultraviolet radiation was decreased by 67.42%. Crack propagation can be controlled to some extent by the addition of natural fillers to polypropylene [31,32,34,35,50,61,62]. Figure 4 show the SEM photographs of iPP composite containing calcium carbonate, raw Opoka, and calcined Opoka unaged and after exposure to UV radiation for 1000 h.

When comparing Figure 3 and Figure 4, it is clearly visible that after 1000 h of accelerated aging, surface microcracks also appeared on the surface of samples containing calcium carbonate (Figure 4B), but they were more superficial (less deep) than in the case of the neat iPP (Figure 3). Scratches and microcracks did not appear on the surfaces of composite samples containing Opoka in their structure, which confirmed the greater resistance of these materials to the accelerated degradation processes. By the introduction of used fillers into the iPP matrix, the extent of material degradation during UV radiation could be reduced. This can be also evidenced from Figure 2B, in which it is clear that the extent of tensile strength degradation for the neat iPP after 1000 h was 67.42%, whereas that for iPP/CC was 54.12, for iPP/RO was 32.64%, and for iPP/CO was 23.56%. Analogous dependencies; i.e., a decrease in the difference between the value of the unaged sample compared to the aged sample, in the order iPP ≤ iPP/CC < iPP/RO < iPP/CO, were also noted for the elongation at break and Shore hardness. Opoka was found to be a superior stabilizer when compared to CaCO_3_. This was partially due to silica content showing UV-blocking properties, as discussed above, but also making the filler more water-resistant. When carefully analyzing the sample surfaces using SEM before and after aging, it could be seen that erosion was visible around the CaCO_3_ particles, as well as some pores in which the filler particles were likely missing (Figure 4B). As the accelerated aging experiment had a condensation dark period (sample water sprinkling), the filler may have been washed off the surface of the sample due to the slight solubility of CaCO_3_ in water. This behavior was not observed for the Opoka-filled samples (Figure 4D,F). This was due to the presence of insoluble SiO_2_ content in Opoka, resulting in the filler particles being less susceptible to moisture influence. Therefore, during aging, some of the polymer on the composite surface was degraded and removed, revealing the insoluble filler particles.

When analyzing the presented SEM images, it is also worth paying attention to the surfaces of unaged composite samples (Figure 4A,C,E). Due to the addition of powder fillers, the surfaces of the composites appeared uneven before aging. However, the difference in the filler-matrix interphase on the surface of the composite was evident; i.e., the calcium carbonate particles seemed to be poorly wetted with the polymer, while the Opoka particles of both grades were covered with a delicate polymer layer, which may also have had a positive effect on limiting the aging process of these composites and stabilization of the filler on the polymer surface.

The presented results clearly indicated that the addition of Opoka to iPP not only contributed to an enhanced tensile modulus and hardness of composites, but also effectively suppressed the loss of mechanical properties during the aging process.

### 3.4. Carbonyl Index

FT-IR is an effective tool to characterize the changes of the molecular structure during the aging process of the samples. Suitable spectral reference peak should not be affected by accelerated aging, and should be completely isolated from other spectral peaks. Accordingly, in this study, the peak at 1454 cm^−1^ associated with C-H bending and stretching was selected as the spectral reference peak [63]. The carbonyl index (CI), defined as the ratio of the peak height at 1720 cm^−1^, assigned to the carbonyl group stretching, to the peak height at 1454 cm^−1^, was used as a parameter to monitor the degree of photo-oxidation of polypropylene and its composites [57,61,63]. The FT-IR spectra of the novel studied fillers were discussed in detail in our previous work [43].

**Figure 5:**
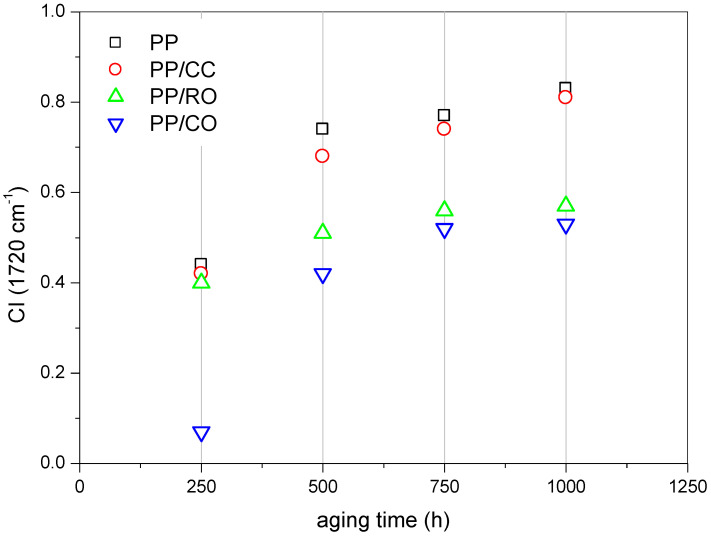
The changes in carbonyl index of iPP and its composites after accelerated aging.

The determined values of the carbonyl index (Figure 5) indicated that the degradation of neat PP proceeded much faster than its composites. This was related to two factors, one being the UV-blocking effect of the fillers, and the second the crystallinity of the samples (see Table 1). UV irradiation of the polymer caused formation of free radicals and chain scission. On the other hand, the kinetics of chain oxidation depended mainly on the diffusion of oxygen into the sample. The high degree of packing of atoms in the crystalline region can significantly limit the gas permeability [64,65]. For neat polypropylene and its composites containing calcium carbonate, the increase in CI reached a similar value (0.44 and 0.42, respectively) after 250 h of accelerated aging, and then gradually increased with the increase in aging time, but with the CaCO_3_-filled composite reaching a lower CI index, however. An analogous observation was made by Liu H. et al. [61], but they suggested that CaCO_3_ particles could uniformly disperse in a polymer matrix and achieve good interfacial adhesion, which contributed to blocking of ultraviolet light from reaching the composite matrix and slowing the aging process. In addition, Yang et al. [31] suggested the shading effect of calcium carbonate, reducing the photo-oxidative degradation of polymeric composites. A different trend was observed for the Opoka-filled composites. For the iPP/RO composite after 250 h of aging, the CI was similar to the above-mentioned composites (0.41), after which the degradation rate dropped significantly. The ∆CI was 0.11, 0.05, and 0.01 at 500 h, 750 h and 1000 h, respectively. For iPP/CO, the CI values for prolonged aging were similar, but after 250 h. it was merely 0.07, suggesting increased aging induction time for the given system. The ∆CI for further aging times was 0.35, 0.1, and 0.01, at 500 h, 750 h, and 1000 h, respectively. The improved stability over the initial aging period might have been due to the high pH of calcined Opoka (discussed in the earlier work [43]), hampering the formation and further reaction of some degradation intermediates, such as carboxylic acid or peroxyl moieties [66,67].

The obtained results of the calculated carbonyl index were consistent with all the relationships presented above, and clearly confirmed the previous conclusions that the Opoka, both in the raw and calcined forms, increased the aging stability of the polypropylene composites.

## 4. Conclusions

In this work, the possibility of using Opoka as a novel filler for polypropylene was investigated. Two different filler grades were used for the study; that is, raw (RO) and calcined (CO) Opoka. Both grades were proven to be compatible with iPP, causing no significant deterioration of mechanical parameters of the resulting composites. Morphology analysis based on SEM imaging showed better filler surface wetting with the polymer for iPP/RO and iPP/CO, when compared to calcium carbonate (iPP/CC). More importantly, the results of accelerated aging proved that Opoka was an effective UV stabilizer, superior to CaCO_3_ in this matter, prolonging the iPP composites’ service life in terms of mechanical properties or potential reprocessing, based on SEM imaging, mechanical tests, DSC studies, and crystallization behavior. Therefore, Opoka may not only be used as a simple extension filler, but especially as a UV blocker/stabilizer for outdoor applications of the iPP composites. On the basis of numerous literature reports, it is known that, depending on the structure, the stability and resistance to photodegradation of polymers varies. Therefore, in order to confirm the properties of the Opoka stabilizing action against UV radiation, as a continuation of the research presented in this article, it is planned to produce composites based on other thermoplastic and biodegradable polymers containing Opoka, and then to assess the impact of accelerated aging on their mechanical and thermal properties.

## Figures and Tables

**Table 1:** Thermal parameters of neat iPP and its composites obtained from DSC.

	*T_m_*_1_/°C	∆*H_m_*_1_	*T_c_*_1_/°C	*X*_1_/%	*T_m_*_2_/°C	∆*H_m_*_2_	*T_c_*_2_/°C	*X*_2_/%
iPP	166.9	68.1	117.3	32.9	163.6	98.9	117.1	47.8
iPP/CC	167.1	72.0	120.8	36.6	162.5	94.8	119.6	48.2
iPP/RO	168.3	73.4	121.5	37.3	164.3	104.0	121.5	52.9
iPP/CO	166.7	75.3	123.4	38.3	162.9	104.2	122.3	53.0

**Table 2:** Thermal properties of the iPP composites (first and second heating cycles) as a function of accelerated aging.

	*T_m_*_1_/°C	*T_c_*_1_/°C	*T_m_*_2_/°C	*T_c_*_2_/°C
iPP	166.9	117.3	163.6	117.1
iPP_250	166.7	117.8	164.4	117.8
iPP_500	162.4	119.0	156.5	117.8
iPP_750	165.6	118.2	160.5	117.3
iPP_1000	163.4	117.9	159.7	117.9
iPP/CC	167.1	120.8	162.5	119.6
iPP/CC_250	161.2	117.9	156.3	116.5
iPP/CC_500	161.9	118.1	157.0	117.3
iPP/CC_750	163.7	116.9	155.7	114.5
iPP/CC_1000	165.6	118.1	157.6	117.7
iPP/RC	168.3	121.5	164.3	121.5
iPP/RC_250	165.0	119.8	160.9	119.3
iPP/RC_500	160.5	119.9	160.0	119.4
iPP/RC_750	166.7	119.6	160.4	119.3
iPP/RC_1000	167.2	119.4	161.6	118.8
iPP/CO	166.7	123.4	162.9	122.3
iPP/CO_250	165.6	121.2	162.2	121.3
iPP/CO_500	162.4	120.3	159.9	119.9
iPP/CO_750	163.4	120.2	159.9	119.4
iPP/CO_1000	164.2	119.7	160.8	118.3

**Table 3:** Mechanical properties of unaged iPP and the obtained composites.

	E_t_, GPa	σ_M_, MPa	ε_B_, %	HS, ^0^Sh
PP	1.29 ± 0.01	32.3 ± 0.41	45.2 ± 3.58	61.8 ± 0.1
PP/CC	1.46 ± 0.01	33.5 ± 0.05	28.6 ± 2.60	62.2 ± 0.1
PP/RO	1.49 ± 0.01	32.6 ± 0.31	24.2 ± 5.73	63.8 ± 0.1
PP/CO	1.38 ± 0.02	32.0 ± 0.35	30.2 ± 5.52	62.1 ± 0.2

E_t_—Young’s modulus, σ_M_—tensile strength, ε_B_—elongation at break, HS—Shore hardness (D scale).

## Data Availability

Not applicable.
